# High- and low-dose cyclophosphamide in Egyptian lupus nephritis patients: a multicenter retrospective analysis

**DOI:** 10.1007/s00393-023-01386-7

**Published:** 2023-08-15

**Authors:** Mohamed Momtaz A. Elaziz, Sherif M. Gamal, Ahmed Fayed, Mohammed Hassan Abu-Zaid, Shada A. Ghoniem, Doaa A. Teleb

**Affiliations:** 1 Department of Internal Medicine, Nephrology Unit, Cairo University Hospital, Cairo, Egypt; 2 Rheumatology and Rehabilitation Department, Faculty of medicine, Cairo University Hospital, Cairo University, Al Kasr Al Aini, Old Cairo, Cairo Governorate, 4240310 Cairo, Egypt; 3 Rheumatology and Rehabilitation Department, Tanta University Hospital, Tanta, Egypt

**Keywords:** Systemic lupus erythematosus, Induction therapy, Renal outcome, Proteinuria, Renal function, Remission, Systemischer Lupus erythematosus, Induktionstherapie, Nierenergebnisse, Proteinurie, Nierenfunktion, Remission

## Abstract

**Background:**

Lupus nephritis (LN) is a common serious presentation of systemic lupus erythematosus. Cyclophosphamide (CYC) and mycophenolate mofetil (MMF) are listed as the first-line drugs in induction therapy for LN.

**Objective:**

This study aimed to compare high- and low-dose CYC in a cohort of Egyptian LN patients.

**Patients and methods:**

The data of 547 patients with class III/IV active LN who received CYC as induction therapy were retrospectively analyzed. Whereas 399 patients received 6‑monthly 0.5–1 g/m^2^ CYC doses, 148 patients received six biweekly 500 mg CYC doses. Demographic data, laboratory test results, and disease activity index were recorded and compared at presentation and at 6, 12, 18, 24, and 48 months of follow-up.

**Results:**

After 48 months, the proportion of patients maintaining normal creatinine levels was higher in the group receiving induction therapy with high-dose CYC (67.9%, 60.4%, *p* = 0.029), and these patients also had higher proteinuria remission at 36 (26.6%, 14.8%, *p* = 0.014) and 48 months (24.3%, 12.8%, *p* = 0.006). Comparison of patient outcomes according to both induction and maintenance therapy showed the best results in patients who received high-dose CYC and continued MMF as maintenance therapy.

**Conclusion:**

High- and low-dose CYC are comparable in early phases of treatment. However, after a longer duration of follow-up, high-dose CYC was associated with higher remission rates in the current cohort.

## Abstract (de)

**Hintergrund:**

Die Lupusnephritis (LN) ist eine der häufigen schwergradigen Manifestationen eines systemischen Lupus erythematosus. Cyclophosphamid (CYC) und Mycophenolatmofetil (MMF) sind als Substanzen der Erstlinienbehandlung für die Induktionstherapie bei LN aufgeführt.

**Ziel:**

Ziel der vorliegenden Studie war es, die hochdosierte und die niedrigdosierte Gabe von CYC in einer Kohorte von ägyptischen LN-Patienten zu untersuchen.

**Patienten und Methoden:**

Dazu wurden die Daten von 547 Patienten mit aktiver LN der Klasse III/IV und Gabe von CYC als Induktionstherapie retrospektiv untersucht. Während 399 Patienten 6 Dosen von 0,5–1 g/m^2^ CYC monatlich erhielten, wurden 148 Patienten mit 6 Dosen 500 mg CYC alle 2 Wochen therapiert. Demografische Daten, Laborergebnisse und Krankheitsaktivitätsindex wurden dokumentiert und bei Erstvorstellung sowie bei den Nachuntersuchungen nach 6, 12, 18, 24 und 48 Monaten verglichen.

**Ergebnisse:**

Nach 48 Monaten war die Anzahl der Patienten mit normal gebliebenem Kreatinin in der Gruppe, die eine Induktionstherapie mit Hoch-Dosis-CYC erhalten hatte höher (67,9 %; 60,4 %; *p* = 0,029), und diese Patienten wiesen auch höhere Werte für die Remission hinsichtlich einer Proteinurie nach 36 (26,6 %; 14,8 %; *p* = 0,014) und 48 Monaten auf (24,3 %; 12;8 %; *p* = 0,006). Der Vergleich in Bezug auf das Outcome der Patienten nach Induktions- und Erhaltungstherapie zeigte die besten Ergebnisse für Patienten, die Hoch-Dosis-CYC erhalten hatten und die Therapie mit MMF als Erhaltungstherapie fortführten.

**Schlussfolgerung:**

Hoch- und Niedrig-Dosis-CYC sind in den frühen Phasen der Behandlung vergleichbar. Jedoch war nach einer längeren Nachbeobachtungsdauer Hoch-Dosis-CYC mit höheren Remissionsraten in der aktuellen Kohorte verbunden.

## Key points

- High- and low-dose CYC are comparable in early phases of treatment.
- High-dose CYC was associated with a higher remission rate after a longer duration of follow-up.

## Introduction

Lupus nephritis (LN) is a common serious presentation of systemic lupus erythematosus (SLE), with varying degrees of glomerular and tubulointerstitial pathology [1, 2]. LN may occur in up to 50% to 60% of patients with SLE and is a major predictor of poor prognosis [2–4]. In 25–50% of lupus patients, it may be one of the presenting manifestations, and although SLE is more common in females, male patients tend to have more severe LN [2, 5].

Cyclophosphamide (CYC) and mycophenolate mofetil (MMF) are listed as the first-line drugs in induction therapy for LN [6]. Mycophenolate mofetil is recommended for first-line treatment of LN for its better safety profile as regards ovarian failure compared to CYC, with “noninferior outcomes” [7]. However, MMF may be less commonly used than CYC (either high- or low-dose) in some countries due to its high cost [6].

The side effects associated with high-dose CYC, especially gonadal toxicity, favor the use of low-dose CYC (Euro-Lupus regimen), as it is associated with fewer side effects and showed comparable efficacy to the high-dose regimen except in association with severe disease and poor prognostic factors including reduced glomerular filtration rate and histologic presence of fibrous crescents, fibrinoid necrosis, or tubular atrophy/interstitial fibrosis. In these conditions, MMF and high-dose CYC show better outcomes [8, 9].

Taking into consideration the previously mentioned preference for CYC use in some countries and the well-documented ethnic impact on both disease presentation and outcomes in LN [10, 11], this study aimed to compare high- and low-dose CYC in a cohort of Egyptian LN patients regarding long-term disease outcomes.

## Materials and methods

### Participants

In 2021 and early 2022, the medical records of SLE patients with biopsy-proven LN who had attended follow-up visits during the past 5 years at three different rheumatology and nephrology centers were retrospectively reviewed. These patients’ information was gathered from the Rheumatology and Rehabilitation Department, Cairo University; the Nephrology Unit of the Internal Medicine Department, Cairo University; and the Rheumatology and Rehabilitation Department, Tanta University. Records were obtained starting January 2016 until January 2021. Lupus nephritis patients were consecutively enrolled, excluding patients lost to follow-up after CYC, those with incomplete data in the medical records, and those who did not complete their induction regimen. Accordingly, 87 patients were excluded.

Systemic lupus erythematosus patients met either the 1997 Modified American College of Rheumatology Criteria for the classification of SLE [12] or the Systemic Lupus International Collaborative Clinics criteria [13]. The presence of increased serum creatinine, proteinuria, and/or active urine sediment was used to diagnose the beginning of nephritis. Hypertension was diagnosed as a sustained rise in blood pressure of 140/90 mmHg or the use of antihypertensive medicines, according to the Eighth Joint National Committee (JNC8) categorization [14].

Patients were divided into two groups based on the treatment regimen:

1. patients in group 1 underwent a low-dose CYC LN protocol consisting of 500 mg intravenous CYC every other week for a total of six doses;
2. patients in group 2 received high-dose CYC, i.e., intravenous CYC (0.5–1 g/m^2^) monthly for 6 months.

### Ethical considerations

The study protocol was modified and approved by the Rheumatology and Rehabilitation Department, Faculty of Medicine, Tanta University, with approval number 34997/10/21. The study adhered to the ethical norms of the 1975 Declaration of Helsinki, as approved by the institution’s human science committee [15]. Privacy of all patient data was granted as there was a code number for every patient file that included all investigations.

### Assessment

Complete blood count (CBC), erythrocyte sedimentation rate (ESR), blood urea, serum creatinine, serum albumin, full urine analysis, 24‑h urinary protein, and serum complement components C3 and C4 were among the laboratory data gathered from the patients. Antinuclear antibodies (ANA), anti-dsDNA antibodies (anti-DNA), anticardiolipin antibodies (aCL), and lupus anticoagulant (LA) were all documented as positive or negative in the patients’ immunological profiles.

To assess disease activity, the Systemic Lupus Erythematosus Disease Activity Index 2000 (SLEDAI-2K) was used [16]. Proteinuria > 0.5 g/day, hematuria and pyuria (both > 5 cells/high-power field), and cellular casts all receive a score of 4 if they are present [17]. All patients’ demographic and clinical data, including age at diagnosis, disease duration, SLE disease activity as defined by SLEDAI-2K, and individual organ-specific disease involvement, laboratory data, and current medications were gathered at the time of visits.

Renal biopsy was performed for SLE patients with proteinuria greater than 0.5 g/day or active urine sediment. Renal biopsy activity was determined using modified National Institutes of Health (NHI) activity and chronicity indices: the NIH Lupus Activity Index (NIH-AI; score of 24) and the NIH Lupus Chronicity Index (NIH-CI; score of 12), both of which were evaluated by a renal pathologist [18].

Induction therapy was given as an initial treatment either by a low-dose CYC LN protocol consisting of 500 mg of intravenous CYC every other week for a total of six doses, or high-dose CYC, which consists of intravenous CYC (0.5–1 g/m^2^) given monthly for 6 months. Both regimens are approved in the three centers of study and both regimens were used interchangeably.

Clinical and laboratory assessment was performed during follow-up visits at 6, 12, 24, 36, and 48 months following the initial treatment.

Other drugs received by the patients included corticosteroids, which were started in all patients with an intravenous methylprednisolone pulse with induction therapy. Subsequently, 1 mg/kg oral prednisone was given with gradual tapering according to clinical response. Standard maintenance prednisone was 5–10 mg for all patients.

Renal remission was defined by inactive urinary sediment, proteinuria < 0.5 g/24 h, and/or trace protein in a random urine sample *and *normal renal function tests [19].

### Statistical methods

All data were entered into a Microsoft Excel (Redmond, WA, USA) spreadsheet. Statistical analysis was done using MedCalc® Statistical Software (MedCalc Software Ltd., Ostend, Belgium) version 20.110 [20]. Continuous data were tested for normal distribution using the D’Agostino–Pearson test [21]. Normal distribution was rejected for most variables. Thus, summary statistics were expressed in terms of minimum, maximum, median, and interquartile range. Nonparametric tests were used. Chi-squared test was used to compare categorical variables (frequencies), while the Mann–Whitney test was used for continuous variables. Multiple comparison was done using the Kruskal–Wallis test (comparison of continuous data among more than two groups). Serial-measurement analysis of variance (ANOVA) was used to compare fixed variables across multiple time intervals. A *p*-value of < 0.05 was considered statistically significant.

## Results

### Patients

The data of 547 patients who had been diagnosed with class III/IV active LN over the past 5 years and received CYC as induction therapy were retrospectively analyzed.

These patients were diagnosed and treated in three different centers: 259 in the Rheumatology and Rehabilitation Department of Cairo University Hospital, 256 patients in the Nephrology Unit of Cairo University Hospital, and 32 patients in the Rheumatology and Rehabilitation Department in Tanta University Hospital.

Of these patients, 399 received 6‑monthly 0.5–1 g/m^2^ CYC doses and 148 patients received six biweekly 500 mg CYC doses.

### Baseline characteristics of patients

There were 509 (93.1%) female patients and 38 male patients (6.9%). The high-dose CYC regimen group included 30 male patients (7.5%) while the low-dose CYC group included 8 male patients (5.4%; not significant, *P* = 0.3882). Hypertension was diagnosed in 145/399 (36.3%) patients who followed the high-dose CYC regimen, but in only 22/148 (14.9%) patients who followed the low-dose biweekly CYC regimen, a difference with high statistical significance (*P* < 0.001). Most of the baseline clinical and laboratory criteria did not significantly differ between the two groups, except that those who received high-dose CYC were older at baseline, had higher SLEDAI scores, higher ESR, and lower baseline C3 levels than those who received low–dose CYC (*P*-values 0.0251, 0.0035, 0.0001, and 0.0007, respectively; Table 1). Regarding patients’ comorbidities, 30.7% of patients were hypotensive, 6.2% were diabetics, and 4.5% had hepatitis C virus antibodies (HCVab; Table 1). Among the baseline renal biopsy pathological findings, patients who received high-dose CYC had higher chronicity indices at baseline (*P*-value 0.0001), whereas those who received low-dose CYC had more thrombotic microangiopathy (TMA) at baseline (*P*-value < 0.0001; Table 2).

**Table 1:** Baseline characteristics of included patients

	Total cohort	High-dose CYC					Low-dose CYC					*P*-value
	%	Min	Max	Median	IQR	%	Min	Max	Median	IQR	%	Mann–Whitney
*Age (years)*	–	14	45	29	22–36	–	16	48	27	22–33	–	0.0251*
*BMI (kg/m*^*2*^*)*	–	19.5	31.6	27.6	25.1–30.9	–	19.5	35.9	26.7	23.9–29.7	–	0.1030
*Duration of SLE (months)*	–	1	32	9	6–15	–	1	21	9	6–12.8	–	0.3415
*Duration of LN (months)*	–	0.75	23	5	3–8	–	1	17	4	3–7	–	0.1521
*Total SLEDAI*	–	1	53	12	8–17.3	–	4	42	11	8–14	–	0.0035*
*Hb (g/dl)*	–	2.7	17	11	9.1–12.9	–	2.7	15.8	10.6	9–13	–	0.3315
*WBCs (x10*^*3*^*/µl)*	–	1.6	18	7.0	5–8.7	–	2.5	17	6.7	5.35–9	–	0.8369
*Platelet (x10*^*3*^*/µl)*	–	5	687	263	190.5–320	–	46	473	238	190–325	–	0.6841
*ESR (mm/h)*	–	8	150	70	44.3–100	–	10	135	54	30–83	–	0.0001*
*ALT U/L*	–	4	291	19	13–26	–	4	170	22	15–26	–	0.2964
*Serum total calcium (mg/dl)*	–	2	10.7	8.9	8.5–9.2	–	6	10.3	9.0	8.6–9.2	–	0.3111
*Serum phosphorous (mg/dl)*	–	1.2	7.9	3.9	3.5–4.2	–	1.9	53	4	3.6–4.3	–	0.1703
*Blood urea (mg/dl)*	–	20	286	49	30–63.75	–	20	290	50	34.5–61	–	0.7104
*Serum creatinine (mg/dl)*	–	0.8	7.5	2.25	2–2.8	–	0.8	6.7	2.45	2–2.8	–	0.3923
*Proteinuria (g/day)*	–	1.1	9.5	3	2–3.6	–	1.1	9	2.9	1.9–3.5	–	0.6219
*Serum uric acid (mg/dl)*	–	1.4	11	5.1	4.4–6.9	–	3	11.6	5	4.5–6.9	–	0.7892
*Serum C3 (mg/dl)*	–	7	199.1	72	59–88.1	–	24	199.1	82	65.7–110.3	–	0.0007*
*Serum C4 (mg/dl)*	–	2	39	8	6.65–14	–	3	39	8	6.2–21.5	–	0.3294
*CRP (mg/l)*	–	0.1	364	2.4	0.92–7.4	–	0.1	25	2	0.5–5	–	0.1914
*ANA positive*	100	–	–	–	–	100	–	–	–	–	100	–
*Anti dsDNA positive*	89.4	–	–	–	–	90.2	–	–	–	–	87.4	0.3540
*Low C3*	32.6	–	–	–	–	36.5	–	–	–	–	77.4	0.0036*
*Low C4*	66.0	–	–	–	–	67.8	–	–	–	–	61.4	0.1830
*Comorbidities*												
Diabetes	6.2	–	–	–	–	7.6	–	–	–	–	2.7	0.0407*
Hypertension	30.7	–	–	–	–	36.5	–	–	–	–	15.1	< 0.0001*
HCVab	4.5	–	–	–	–	3.2	–	–	–	–	7.7	0.0263

*CYC* cyclophosphamide, *BMI* body mass index, *LN* lupus nephritis, *SLEDAI* Systemic Lupus Erythematosus Disease Activity Index, *Hb* hemoglobin, *WBC* white blood cell count, *ESR* erythrocyte sedimentation rate, *ALT* alanine aminotransferase, *AST* aspartate aminotransferase, *C* complement, *CRP,* C‑reactive protein, *ANA* antinuclear antibodies, *AntidsDNA* anti double-stranded DNA, *HCVab* hepatitis C virus antibodies, *IQR *interquartile range

*P-value is significant (< 0.05)

**Table 2:** Renal biopsy findings of included patients

Variable	High-dose cyclophosphamide (%)	Low-dose cyclophosphamide (%)	*P*-value
*Activity index/12*			
≤ 12	88.7	90.8	0.5512
> 12	11.3	9.2	
*Chronicity index/12*			
0	50.3	75.3	0.0001*
1	27.5	22.5	
2	14.4	2.2	
3	7.8	0.0	
*TMA*			
No	72.3	49.6	< 0.0001*
Yes	27.7	50.4	
*Lupus vasculopathy*			
No	93.7	94.7	0.7032
Yes	6.3	5.3	
*Vasculitis*			
No	91.0	96.2	0.0535
Yes	9.0	3.8	
*Intimal fibrosis*			
No	89.5	90.2	0.8105
Yes	10.5	9.8	

*TMA* thrombotic microangiopathy

*P-value is significant (< 0.05)

There was no significant difference between the groups as regarding the baseline-associated autoantibodies ANA, anti-DNA, aCL IgM, IgG, and LA.

### Comparison of patient outcomes according to induction therapy

After 6 months of treatment, a normal level of serum creatinine was achieved in comparable percentages of patients receiving induction therapy with either high- or low-dose CYC (75.2%, 70.9%, *p* = 0.351). There was also no difference between the regimens regarding the persistence of remission of serum creatinine at 12, 18, and 36 months from the start of treatment. However, after 48 months, the percentage of patients who remained in remission was higher in the group who had received induction therapy by high-dose monthly CYC (67.9%, 60.4%, *p* = 0.029; Table 3).

**Table 3:** Percentage of patients achieving normal serum creatinine and remission of proteinuria at various time intervals according to induction therapy

Variable	Remission of serum creatinine			Remission of proteinuria		
	High dose CYC *N* = 399 (%)	Low dose CYC *N* = 148 (%)	*P*-value ^a^	High dose CYC *N* = 399 (%)	Low dose CYC *N* = 148 (%)	*P*-value^a^
6‑month	300 (75.2%)	105 (70.9%)	0.351	84 (21%)	19 (12.8%)	0.033*
12-month	323 (80.9%)	114 (77.0%)	0.307	128 (32.1%)	39 (26.3%)	0.196
18-month	304 (76.2%)	101 (68.2%)	0.091	113 (28.3%)	27 (18.2%)	0.106
36-month	295 (73.9%)	98 (66.2%)	0.142	106 (26.6%)	22 (14.8%)	0.014*
48-month	271 (67.9%)	89 (60.4%)	0.029*	97 (24.3%)	19 (12.8%)	0.006*

Remission of proteinuria: patients achieving proteinuria < 0.5 g

^a^Chi-squared test

*P-value is significant (< 0.05)

After 12 and 18 months from starting treatment, urinary protein excretion was reduced to < 0.5 g per day in comparable percentages of patients in both regimens, with a *p*-value of 0.196 and 0.106, respectively. However, after a longer duration of follow-up, the monthly high-dose CYC induction regimen was associated with a higher percentage of patients retaining proteinuria remission: 26.6% and 14.8% (*p* = 0.014) after 36 months and 24.3% and 12.8% (*p* = 0.006) after 48 months (Table 3).

### Comparison of patient outcomes according to induction and maintenance therapy

Patients received as maintenance either azathioprine (34.6%; 30.8% of the classic regimen group and 44.6% of the Euro regimen group) or MMF (65.4%; 69.2% of the classic regimen group and 55.4% of the Euro regimen group) combined with hydroxychloroquine and variable doses of corticosteroids.

For better analysis considering the effect of both induction and maintenance treatment, the involved patients were further categorized according to the administration of MMF or azathioprine (AZA) as maintenance therapy after either of the two induction regimens.

The percentage of patients achieving normal serum creatinine was significantly different when patients were compared according to induction and maintenance therapy at 12, 18, 36, and 48 months (*p* = 0.0175, 0.0006, 0.0452, and 0.0414, respectively). Best results were seen in patients who received high-dose monthly CYC and continued MMF maintenance at all time intervals (Table 4**)**.

**Table 4:** Percentage of patients achieving normal serum creatinine at various time intervals according to both induction and maintenance therapy

Variable	1. High-dose monthly CYC + MMF *N* = 276 (%)	2. High-dose monthly CYC + AZA *N* = 123 (%)	3. Low-dose biweekly CYC + MMF *N* = 82 (%)	4. Low-dose biweekly CYC + AZA *N* = 66 (%)	*P*-value^a^	Significant subgroups^a^
12-month	209 (85.0%)	76 (71.7%)	53 (73.6%)	46 (80.7%)	0.110558	–
18-month	179 (78.5%)	57 (57.6%)	42 (64.6%)	34 (63.0%)	0.043595*	1 vs. 2
36-month	163 (79.9%)	60 (66.7%)	38 (66.7%)	34 (70.8%)	0.266912	–
48-month	146 (81.1%)	56 (70.9%)	34 (64.2%)	30 (69.8%)	0.104266	–

*CYC* cyclophosphamide, *MMF* mycophenolate mofetil, *AZA* azathioprine

^a^Kruskal–Wallis test

*P-value is significant (< 0.05)

Similarly, the percentage of patients achieving proteinuria < 0.5 g was significantly different when patients were compared according to induction and maintenance therapy at 12, 18, 36, and 48 months. Best results were seen in patients who received high-dose monthly CYC and continued MMF maintenance at all time intervals (Table 5).

**Table 5:** Percentage of patients achieving proteinuria < 0.5 g at various time intervals according to both induction and maintenance therapy

Variable	1. High-dose monthly CYC + MMF *N* = 276 (%)	2. High-dose monthly CYC + AZA *N* = 123 (%)	3. Low-dose biweekly CYC + MMF *N* = 82 (%)	4. Low-dose biweekly CYC + AZA *N* = 66 (%)	*P*-value^a^	Significant subgroups^a^
12-month	93 (38.0%)	19 (18.4%)	20 (28.6%)	13 (22.8%)	0.337929	–
18-month	71 (31.1%)	13 (13.1%)	13 (20.0%)	9 (16.4%)	0.323876	–
36-month	67 (33.2%)	10 (11.2%)	9 (16.1%)	6 (12.8%)	0.000404*	1 vs. 2 1 vs. 4
48-month	63 (35.2%)	5 (6.2%)	8 (15.1%)	4 (9.3%)	< 0.000001*	1 vs. 2 1 vs. 3

*CYC* cyclophosphamide, *MMF* mycophenolate mofetil, *AZA* azathioprine

^a^Kruskal Wallis test

*P-value is significant (< 0.05)

The absolute values of follow-up laboratory results are summarized in Table 6**.**

**Table 6:** Laboratory values at the end of the study

	High-dose CYC				Low-dose CYC				*P*-value (Mann–Whitney)
	Min	Max	Median	IQR	Min	Max	Median	IQR	
*Laboratory values after 36 months*									
Serum C3 (mg/dl)	37.000	187.000	115.000	98.000 to 137.000	77.000	176.000	121.000	111.000 to 143.000	0.2265
Serum C4 (mg/dl)	2.000	45.000	18.500	14.000 to 23.000	9.500	38.000	22.000	14.250 to 25.500	0.1124
Serum creatinine (mg/dl)	0.000	17.000	0.900	0.700 to 1.100	0.400	6.900	0.950	0.800 to 1.300	0.0381*
Proteinuria (g/day)	0.000	26.000	0.800	0.400 to 1.000	0.0300	4.000	0.800	0.500 to 1.000	0.7836
*Laboratory values after 48 months*									
Serum C3 (mg/dl)	12.000	187.000	117.000	97.300 to 137.000	91.000	176.000	123.000	112.000 to 145.000	0.0448*
Serum C4 (mg/dl)	1.000	40.000	22.000	14.000 to 25.500	11.000	40.000	22.000	16.750 to 28.700	0.0757
Serum creatinine (mg/dl)	0.400	6.800	0.900	0.700 to 1.100	0.300	4.800	1.000	0.800 to 1.300	0.0754
Proteinuria (g/day)	0.000	4.800	0.800	0.400 to 1.000	0.0500	1.400	0.800	0.500 to 0.900	0.9269

*IQR* interquartile range

A multivariable analysis model was applied using logistic regression (“enter” method) to test the effect of potential baseline prognostic markers that showed significant differences between the two groups in Tables 1 and 2 in terms of renal remission at different time intervals with NO statistical significance. The results of the multivariable analysis model at the end of the study, at 48 months, are shown in Table 7.

**Table 7:** Multivariable analysis showing the impact of potential confounding variables on long-term remission at the end of the study (48 months)

Variable	Coefficient	Std. error	Odds ratio	95% CI	*P*-value^a^
Age	0.024116	0.022093	1.0244	0.9810 to 1.0697	0.2750
TOTAL SLEDAI	0.016293	0.023078	1.0164	0.9715 to 1.0635	0.4802
ESR	0.0085863	0.0049819	1.0086	0.9988 to 1.0185	0.0848
C3	−0.0064968	0.0042178	0.9935	0.9853 to 1.0018	0.1235
DM	−0.90522	0.82006	0.4045	0.0811 to 2.0180	0.2697
HTN	0.70281	0.44845	2.0194	0.8385 to 4.8636	0.1171
HCV	0.47984	0.53700	1.6158	0.5640 to 4.6291	0.3716
Chronicity index	−0.15488	0.20285	0.8565	0.5755 to 1.2747	0.4452
CYC dose	0.00881	0.43435	1.0088	0.4306 to 2.3635	0.9838
TMA	−0.44667	0.36657	0.6398	0.3119 to 1.3123	0.2230

*SLEDAI* Systemic Lupus Erythematosus Disease Activity Index, *ESR* erythrocyte sedimentation rate, *C3* complement component C3, *DM* Diabetes mellitus, *HTN* Hypertension, *HCV* Hepatitis C virus, *CYC* cyclophosphamide, *TMA* thrombotic microangiopathy, *CI *confidence interval

^a^Multivariable logistic regression, “enter” method

## Discussion

Nephritis is pivotal in the determination of morbidity and mortality in SLE patients. Although CYC has been used successively for decades in the induction of remission, its toxicities remain an issue of major concern [22]. The low-dose CYC regimen or what is known as the Euro regimen was proposed as an alternative to the usual high dose or the National Institute of Health (NIH) regimen, with comparable effectiveness and less detrimental consequences. The current study is one of very few studies from Egypt comparing high- and low-dose CYC regarding renal outcomes at intervals with a follow-up period of 4 years, which we hope will add to the scanty available data from African and Arab countries on this important topic.

The present results showed that there was no difference in renal outcome between the two groups at 6, 12, and 18 months of follow-up. However, at 36 and 48 weeks, the 6‑monthly high-dose group showed better remission results.

These results are similar to those of the study by Mehra et al., where the authors found that at 52 weeks, the high-dose arm had significantly more study subjects with complete/partial response compared to the low-dose group [23]. In parallel, a small retrospective study from Puerto Rico concluded that high-dose CYC therapy is more effective than the low-dose regimen [4]. Nevertheless, the Euro Lupus Nephritis Trial (ELNT) stated that there was no difference in renal outcome between patients with a low-dose intravenous CYC regimen compared to those on a high-dose regimen in a follow-up period of 5 years [24] which was updated in 2010 to a follow-up period of 10 years [25]. Similarly, an earlier study from Egypt showed that the results were comparable in both groups [26]; however, the follow-up period was only 1 year, so the long-term outcomes could not be compared. Another study from Egypt compared high-dose CYC, low-dose CYC, and MMF as induction treatment in proliferative LN. It was found that high-dose CYC shows a better and rapid complete response after the sixth month of treatment in both adults and juvenile LN patients, but after the first year of therapy, the three regimens have comparable efficacy and safety [27]. A study from Japan compared four different therapeutic regimens as induction treatment in SLE nephritis: monthly intravenous CYC, the ELNT protocol, tacrolimus (TAC), or MMF. This study showed no difference in terms of renal response and relapse rates between the four regimens after 3 years of follow-up [28].

Although several studies have compared low-dose CYC and high-dose CYC, some of these studies [29–33] vary greatly regarding not only the definition of high dose and low dose, but also in terms of patient characteristics, the duration of treatment, and the follow-up period. Moreover, the steroids used differ vastly in terms of dose and duration, thus rendering comparison between different studies extremely difficult.

The American College of Rheumatology (ACR) guidelines for management of LN published in 2012 recommended the Euro-Lupus regimen for white patients since no studies involving other ethnic groups were available [34]. The Joint European League Against Rheumatism and European Renal Association—European Dialysis and Transplant Association (EULAR/ERA-EDTA) recommended initiating treatment with MMF or the Euro-Lupus regimen regardless of the ethnicity. They resorted to the high-dose CYC regimen only in patients at a high risk of renal failure, reduced GFR, histological presence of crescents or fibrinoid necrosis, or severe interstitial inflammation [35].

Ethnicity plays a major role in defining the phenotype of SLE and predicting prognosis and mortality. Caucasians tend to have less prevalent nephritis than African Americans, Hispanics, and Asians. In addition, Caucasians show milder disease manifestations throughout the disease course [9]. Studies comparing the various management regimens of LN have rarely involved Caucasians. As mentioned earlier, international recommendations for management did not specify particular advice for the Caucasian ethnicity due to the lack of studies including this ethnic group. While the current results show comparable efficiency of both high- and low-dose CYC apart from the superiority of the high-dose regimen in the long-term follow-up period, strong evidence for the choice of induction regimen in Caucasians is not provided. Randomized controlled trials are crucial to offer a better understanding in this ethnic group. Regarding maintenance therapy, the current study compared patients maintained on AZA to those maintained on MMF. It was deduced that at all time intervals, MMF was superior to AZA in maintaining remission. In accordance with the current results, Dooley et al. found that MMF was better than AZA with respect to time to treatment failure and time to renal flare [36]. Another study by Feng et al. showed that the MMF group not only had a better remission rate and fewer relapses, but that patients on MMF as maintenance therapy also continued to improve throughout the study timeline. These findings were consistent regardless of the induction therapy and other disease and patient characteristics [37]. Consensually, two meta-analyses gave the same conclusion, confirming the superiority of MMF over AZA in maintenance of remission in SLE nephritis [38, 39].

However, other studies concluded both regimens to be equally efficient, with no significant differences in relapse rates. In the MAINTAIN study, Houssiau et al. did not observe any differences in outcome between MMF and AZA, although the authors noted that leucopenia was more frequent in the AZA group [40]. Also, during long-term follow-up after 10 years, the results were the same [41]. ACR guidelines for management of LN do not recommend MMF over AZA, leaving the choice to the physician [34], while the European League Against Rheumatism (EULAR) and European Renal Association-European Dialysis and Transplant Association (EULAR/ERA–EDTA) recommendations give preference to MMF when induction is successfully achieved by MMF in the first place. This recommendation is based on studies showing increased relapse when MMF induction is followed by AZA as maintenance [36]. On the other hand, if pregnancy is planned or the cost of MMF represents a burden, the EULAR/ERA–EDTA recommendations favor AZA over MMF. MMF is costly and cannot be afforded by many patients in a developing country, rendering AZA a first choice in the maintenance therapy of SLE nephritis in clinical settings.

One of the limitations of the current study is that there were some differences in patient characteristics between the groups. For example, patients who received the high-dose regimen had a higher chronicity index, while patients who received the low-dose regimen had more TMA. These differences may affect the outcome. Also due to the retrospective nature of the study, the cumulative steroid dose was not recorded in some patients. More prospective research with adequate follow-up is recommended with the same ethnicity and homogenous characteristics between groups.

Since its first use in the 1970s, CYC has proven to be a gamechanger in SLE, giving hope to physicians before patients of being the ideal treatment for nephritis. However, concerns of safety shattered this idealism. After decades of studying, questions of how much, how long, and when to stop remain issues of debate. Further studies are needed to give our SLE nephritis patients the perfect blend of efficacy and safety.

## Conclusion

High- and low-dose CYC are comparable at early phases of treatment. However, after a longer duration of follow-up, high-dose CYC was associated with higher remission rates in the present cohort.

## Declarations

*Compliance with ethical standards:* All steps were performed according to the revised ethical principles of the Declaration of Helsinki in 2000, and local ethical and methodological protocols for approval of the study were followed. *Ethics approval and consent to participate: *This study is in agreement with the ethical guidelines of the Declaration of Helsinki and it follows the ethical standards of the Tanta Faculty of Medicine, with the institutional ethics board approval number 34997/10/21. Privacy of all patient data was granted, as there was a code number for every patient’s file that included all investigations. *Consent for publication: *The final manuscript has been read and approved by all authors, they have obtained the required ethical approvals, they have given the necessary attention to ensure the integrity of the work, and agreed to publish this work.
